# Benefit of Adjuvant Chemoradiotherapy in Resected Gallbladder Carcinoma

**DOI:** 10.1038/s41598-019-48099-z

**Published:** 2019-08-13

**Authors:** Tae Hyun Kim, Sang Myung Woo, Woo Jin Lee, Eun Sang Oh, Sang Hee Youn, Sung Ho Moon, Sang Soo Kim, Sung Sik Han, Sang-Jae Park, Dae Yong Kim

**Affiliations:** 1 Center for Liver and Pancreatobiliary Cancer, Research Institute and Hospital, National Cancer Center, Goyang, 10408 Korea; 2 Center for Proton Therapy, Research Institute and Hospital, National Cancer Center, Goyang, 10408 Korea

**Keywords:** Gall bladder cancer, Gall bladder cancer

## Abstract

To evaluate the benefit of adjuvant treatments, such as chemoradiotherapy (CRT) and chemotherapy (CTx), compared with no adjuvant treatment (No-AT) in resected gallbladder (GB) cancer patients, 151 patients were analyzed: 98 (64.9%) patients received adjuvant treatment with CRT (n = 59, 39.1%) or CTx (n = 39, 25.8%), and the remaining 53 (35.1%) did not (No-AT). The clinicopathological factors, patterns of failure, locoregional recurrence-free survival (LRFS), recurrence-free survival (RFS) and overall survival (OS) were compared among the three groups according to tumor stage. In patients with T2-3N0M0 stage disease, the incidences of locoregional recurrence and distant recurrence and 5-year LRFS, RFS and OS rates were not significantly different among the No-AT, CTx, and CRT groups (*p* > 0.05 each). In those with T2-3N1-2M0 stage disease, the incidences of locoregional recurrence (11.4%, 78.1%, and 68.4%, respectively) and distant recurrence (42.8%, 73.9% and 66.7%, respectively) in the CRT group were significantly lower than those in the No-AT and CTx groups (*p* < 0.05), and the CRT group had significantly higher 5-year LRFS (82,1%, 26.8%, and 19.0%), RFS (53.3%, 11.6% and 16.7%) and OS rates (64.0%, 22.7% and 4.3%) than the CTx and No-AT groups (*p* < 0.05 each). Therefore, adjuvant CRT may improve the LRFS and RFS and subsequently improve OS in lymph node-positive resected GB cancer.

## Introduction

Gallbladder (GB) cancer is characterized by its rarity, advanced stage at diagnosis despite advances in hepatobiliary imaging techniques and poor prognosis due to aggressiveness in the course of disease. Surgical resection is the only potentially curative treatment for GB cancer, but even after complete resection, locoregional and/or distant recurrences frequently become main problems^1–6^. Conceptually, additional adjuvant treatment modalities, such as chemoradiotherapy (CRT) and chemotherapy (CTx), are required to decrease both locoregional and distant recurrences and subsequently improve survival in patients with GB cancer undergoing surgical resection^5,7–23^. However, the role of adjuvant treatments and optimal treatment modalities remains controversial. Most previous studies^1–4,7–9,14^ regarding adjuvant treatments for GB cancer have included heterogeneous populations that mix GB cancer with extra- and intra-hepatic bile duct cancer as well as patients who have undergone surgical resection with non-curative intent, such as positive resection margin(s) or gross residual tumor. Because of the low incidence of GB cancer, prospective clinical studies to investigate the role and compare the effectiveness of different adjuvant therapeutic modalities, such as CRT and CTx, for GB cancer are difficult to conduct and have rarely been performed. Therefore, further clinical studies to assess the role and compare the effectiveness of different adjuvant modalities in GB cancer patients who have received surgical resection should be needed even with retrospective studies.

The aim of the present study was to evaluate the benefit of adjuvant treatments, such as CRT and CTx, compared with that of surgery alone in resected GB cancer patients.

## Results

A total of 193 patients who received surgical resection for gallbladder cancer from June 2012 to April 2017 were registered. Of these, 42 patients did not meet the eligibility criteria for the following reasons: 21 had T1 disease, 7 had distant metastases, 6 had disease with other histologic types, 3 had other uncontrolled malignancies, 3 underwent R1 resection or palliative resection, and 2 survived fewer than 2 months due to postoperative morbidities. The remaining 151 patients who met all of the inclusion criteria were analyzed in this study. Of these, 98 (64.9%) patients received adjuvant therapy with CTx (n = 39, 25.8%) or CRT (n = 59, 39.1%), and the remaining 53 (35.1%) did not (no adjuvant therapy [No-AT]) (Table 1). In the CTx group, a median of 6 cycles (range, 1–8) of chemotherapy with gemcitabine (n = 31, enrolled in the Phase II adjuvant trial [NCCTS07411]), gemcitabine and cisplatin (n = 5), capecitabine and cisplatin (n = 2), and 5-fluorouracil (5-FU) and leucovorin (n = 1), were received. In the CRT group, a median radiation dose of 50.4 Gy (range, 45–50.4 Gy), 1.8 Gy/fraction was delivered to the planning target volume (PTV) using three-dimensional conformal radiotherapy techniques with four or five coplanar beams of 15 MV photons, and concomitant 5-FU-based chemotherapy was administered. The clinical target volume (CTV) included regional lymph nodes, including the porta hepatis, peripancreatic, celiac, origin of the superior mesenteric artery and para-aortic nodes, with or without a primary tumor bed, and the PTV was defined as the CTV plus 10–20 mm in the craniocaudal direction and 7–10 mm in the other directions to compensate for the setup- and respiration-related uncertainties. The baseline clinicopathologic characteristics of the three groups are summarized in Table 1. Compared with the CTx and CRT groups, the No-AT group had significantly higher frequencies of older age (>60 years) in patients at the T2-3N0M0 stage and significantly higher frequencies of older age (>60 years) and N2 disease in those at the T2-3N1-2M0 stage, respectively (*p* < 0.05 each) (Table 1). The other clinicopathologic characteristics were not significantly different among the three groups (Table 1). The N classification increased as the T classification increased: in 93 patients with T2, N0 was found in 51 (54.8%) patients, N1 in 32 (34.4%) patients, and N2 in 10 (10.8%) patients, and in 58 patients with T3, N0 was found in 18 (31%) patients, N1 in 25 (43.1%) patients, and N2 in 15 (25.9%) patients (*p* = 0.07).

**Table 1:** Comparison of clinicopathologic characteristics according to adjuvant therapy in patients with (i) T2-3N0M0 and (ii) T2-3N1-2M0 stage disease.

Characteristic		(i) T2-3N0M0				(ii) T2-3N1-2M0			
		No-AT (n = 30)	CTx (n = 15)	CRT (n = 24)	*p*	No-AT(n = 23)	CTx (n = 24)	CRT (n = 35)	*p*
Gender	Male	14 (43.5)	8 (53.3)	12 (50.0)	0.952^†^	10 (43.5)	11 (45.8)	13 (37.1)	0.812^†^
	Female	16 (56.5)	7 (46.7)	12 (50.0)		13 (56.5)	13 (54.2)	22 (62.9)	
Age (years)	Median (range)	70 (54–83)	61 (43–76)	64 (45–77)	0.006^‡^	70 (54–86)	64 (38–81)	63 (38–78)	0.007^‡^
	≤60	3 (10.0)	7 (46.7)	9 (37.5)	0.013^†^	3 (13.0)	11 (45.8)	14 (40.0)	0.033^†^
	>60	27 (90.0)	8 (53.3)	15 (62.5)		20 (87.0)	13 (54.2)	21 (60.0)	
Tumor size^*^ (cm)	Median (range)	3.0 (0.1–8.5)	4 (1.0–9.5)	2.5 (0.5–8.0)	0.078^‡^	3.8 (1.6–13)	3.5 (0.9–13.0)	3 (1.0–9.0)	0.199
	≤3	16 (53.3)	4 (26.7)	16 (66.7)	0.053^†^	9 (39.1)	12 (50.0)	18 (51.4)	0.665^†^
	>3	14 (46.7)	11 (73.3)	8 (33.3)		14 (60.9)	12 (50.0)	17 (48.6)	
T classification	T2	24 (80.0)	8 (53.3)	19 (79.2)	0.171^†^	12 (52.2)	10 (41.7)	20 (57.1)	0.500^†^
	T3	6 (20.0)	7 (46.7)	5 (20.8)		11 (47.8)	14 (58.3)	15 (42.9)	
N classification	N1	—	—	—	—	11 (47.8)	18 (75.1)	28 (80.0)	0.032^†^
	N2	—	—	—		12 (52.2)	6 (25.0)	7 (20.0)	
Total no. of dissected LNs	Median (range)	7 (1–15)	10 (0–60)	8 (0–24)	0.155^‡^	11 (1–27)	8.5 (3–47)	10 (1–36)	0.908^‡^
Preop CA 19-9 level	Median (range)	9.9 (2.0–435)	16.8 (2–3100)	15.6 (2–127)	0.191^‡^	28.7 (2–2960)	33.4 (2–8340)	14.4 (5–1969)	0.413^‡^
(U/mL)	≤37	26 (86.7)	11 (73.3)	20 (83.3)	0.603^†^	13 (56.5)	14 (58.3)	28 (80.0)	0.089^†^
	>37	4 (13.3)	4 (26.7)	4 (16.7)		10 (43.5)	10 (41.7)	7 (20.0)	
Postop CA 19-9 level	Median (range)	7.9 (3.5–69.9)	15.0 (2–367)	9.7 (5–49.1)	0.071^‡^	17.5 (2–2960)	20.2 (5–850)	14.2 (4.9–696)	0.382^‡^
(U/mL)	≤37	27 (90.0)	13 (86.7)	22 (91.7)	0.886^†^	17 (73.9)	16 (66.7)	30 (85.7)	0.210^†^
	>37	3 (10.0)	2 (13.3)	2 (8.3)		6 (26.1)	8 (33.3)	5 (14.3)	
Histologic differentiation	WD/MD	22 (73.3)	9 (60.0)	19 (79.2)	0.476^†^	13 (56.5)	17 (70.8)	21 (60.0)	0.598^†^
	PD	8 (26.7)	6 (40.0)	5 (20.8)		10 (43.5)	7 (29.2)	14 (40.0)	
Resection margin	Negative	29 (96.7)	15 (100)	22 (91.7)	0.595^†^	23 (100)	23 (95.8)	34 (97.1)	1.000^†^
	Close	1 (3.3)	0 (0)	2 (8.3)		0 (0)	1 (4.2)	1 (2.9)	
Vascular invasion	No	26 (86.7)	12 (80.0)	22 (91.7)	0.525^†^	11 (47.8)	8 (33.3)	19 (54.3)	0.304^†^
	Yes	4 (13.3)	3 (20.0)	2 (8.3)		12 (52.2)	16 (66.7)	16 (45.7)	
Lymphatic invasion	No	20 (66.7)	7 (46.7)	17 (70.8)	0.278^†^	5 (21.7)	1 (4.2)	12 (34.3)	0.014^†^
	Yes	10 (33.3)	8 (53.3)	7 (29.2)		18 (78.3)	23 (95.8)	23 (65.7)	
Perineural invasion	No	20 (66.7)	7 (46.7)	17 (70.8)	0.278^†^	9 (39.1)	8 (33.3)	13 (37.1)	0.917^†^
	Yes	10 (33.3)	8 (53.3)	7 (29.2)		14 (60.9)	16 (66.7)	22 (62.9)	

Abbreviations: Preop, preoperative; Postop, postoperative; no., number; LN, lymph nodes, CA 19-9, carbohydrate antigen 19-9; WD, well differentiation; MD, moderate differentiation; PD, poorly differentiation; No-AT, no adjuvant therapy; CTx, chemotherapy; and CRT, chemoradiotherapy.

^*^Maximum diameter of the primary tumor, ^†^Fisher’s exact test, ^‡^One-way analysis of variance.

**Figure 1:**
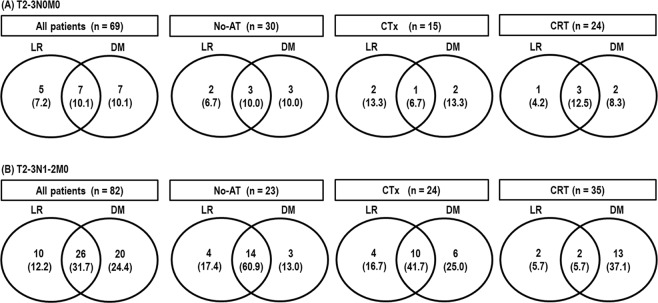
Patterns of failure. Abbreviations: L.R., locoregional recurrence; D.M., distant metastasis; No-AT, no adjuvant therapy; CTx, chemotherapy; and CRT, chemoradiotherapy.

At the time of analysis, 74 patients were alive, and 77 died because of disease progression (n = 69), unknown cause (n = 3), pneumonia (n = 2), underlying liver disease (n = 1), underlying obstructive lung disease (n = 1) and liver abscess (n = 1). The median follow-up time of all patients and living patients was 42.7 months (range, 3.0–207.5 months) and 43.4 months (range, 14.1–207.5 months), respectively. Disease recurrence occurred in 75 (50.3%) patients: 48 (64%) patients had locoregional recurrence, 60 (80%) had distant metastasis, and 33 (44%) had both locoregional recurrence and distant metastasis (Fig. 1). In patients at the T2-3N0M0 stage, locoregional recurrence occurred in 16.7%, 20% and 16.7% of the No-AT, CTx and CRT groups, respectively (*p* = 1.000), and distant metastasis occurred in 20%, 20% and 21.8%, respectively (*p* = 1.000) (Fig. 1A). In patients at the T2-3N1-2M0 stage, locoregional recurrence occurred in 78.1%, 68.4% and 11.4% of the No-AT, CTx, and CRT groups, respectively (*p* < 0.001), and distant metastasis occurred in 73.9%, 66.7% and 42.8%, respectively (*p* = 0.044) (Fig. 1B). In all patients, the median OS time was 81.9 months (95% confidence interval [CI], 13.1–150.8 months), and the 5-year LRFS, RFS and OS rates were 61.8% (95% CI, 53.6–70.0%), 49.7% (95% CI, 41.5–57.9%) and 53.6% (95% CI, 45.4–61.8%), respectively. In patients at the T2-3N0M0 stage and T2-3N1-2M0 stage, the 5-year LRFS, RFS, and OS rates were 78.0% (95% CI, 67.6–88.4%) and 47.6% (95% CI, 36.0–59.2%) (*p* < 0.001), 72.7% (95% CI, 61.9–83.5%) and 30.8% (95% CI, 20.6–41.0%) (*p* < 0.001), and 76.7% (95% CI, 66.3–87.1%) and 34.8% (95% CI, 24.2–45.3%) (*p* < 0.001), respectively.

**Figure 2:**
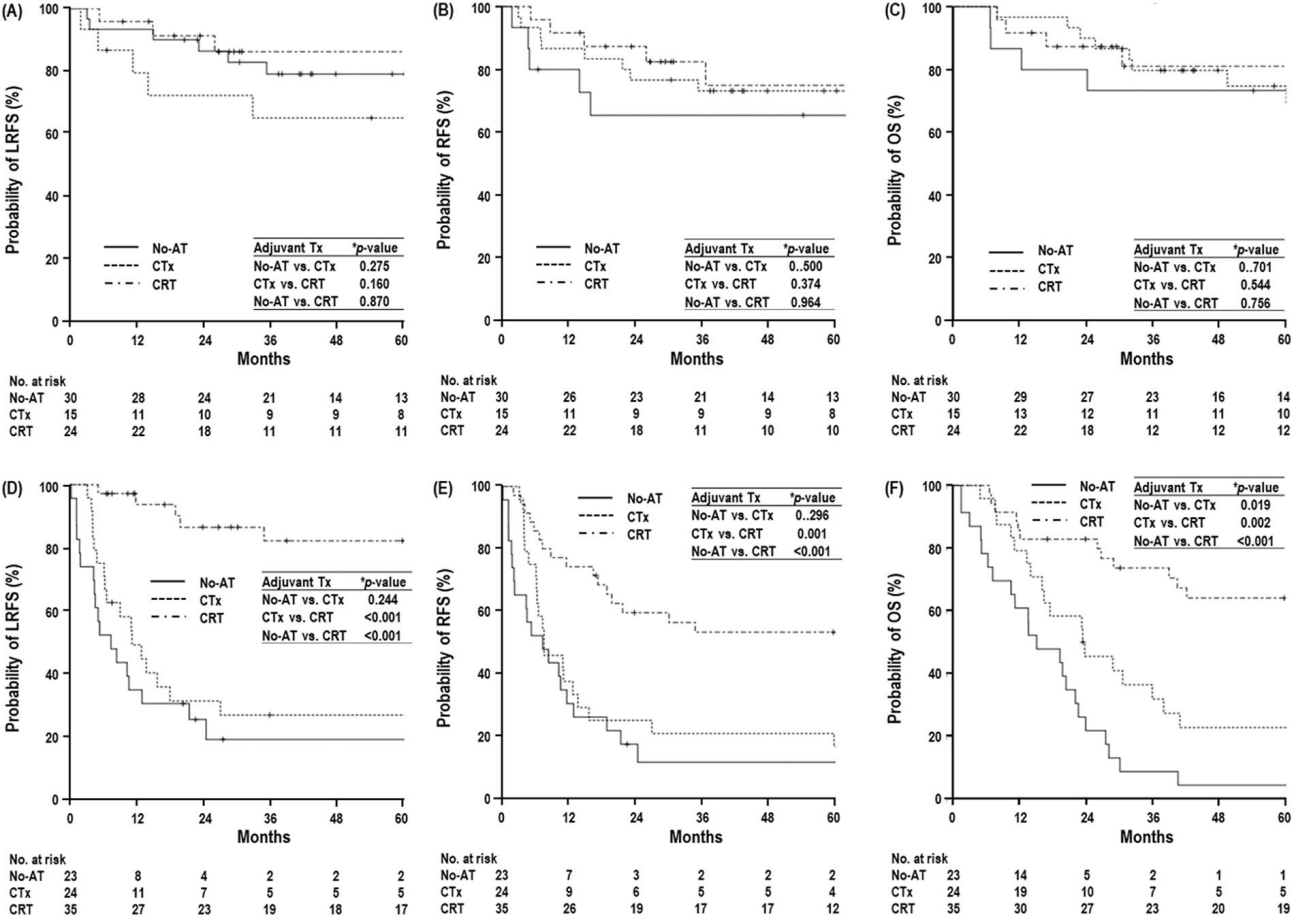
Locoregional recurrence free survival (LRFS), relapse-free survival (RFS), and overall survival (OS) curves according to adjuvant treatments in patients at the T2-3N0M0 stage (**A–C**, respectively) and T2-3N1-2M0 stage (**D–F**, respectively). Abbreviations: same as in Fig. 1. ^*^Log-rank test.

**Figure 3:**
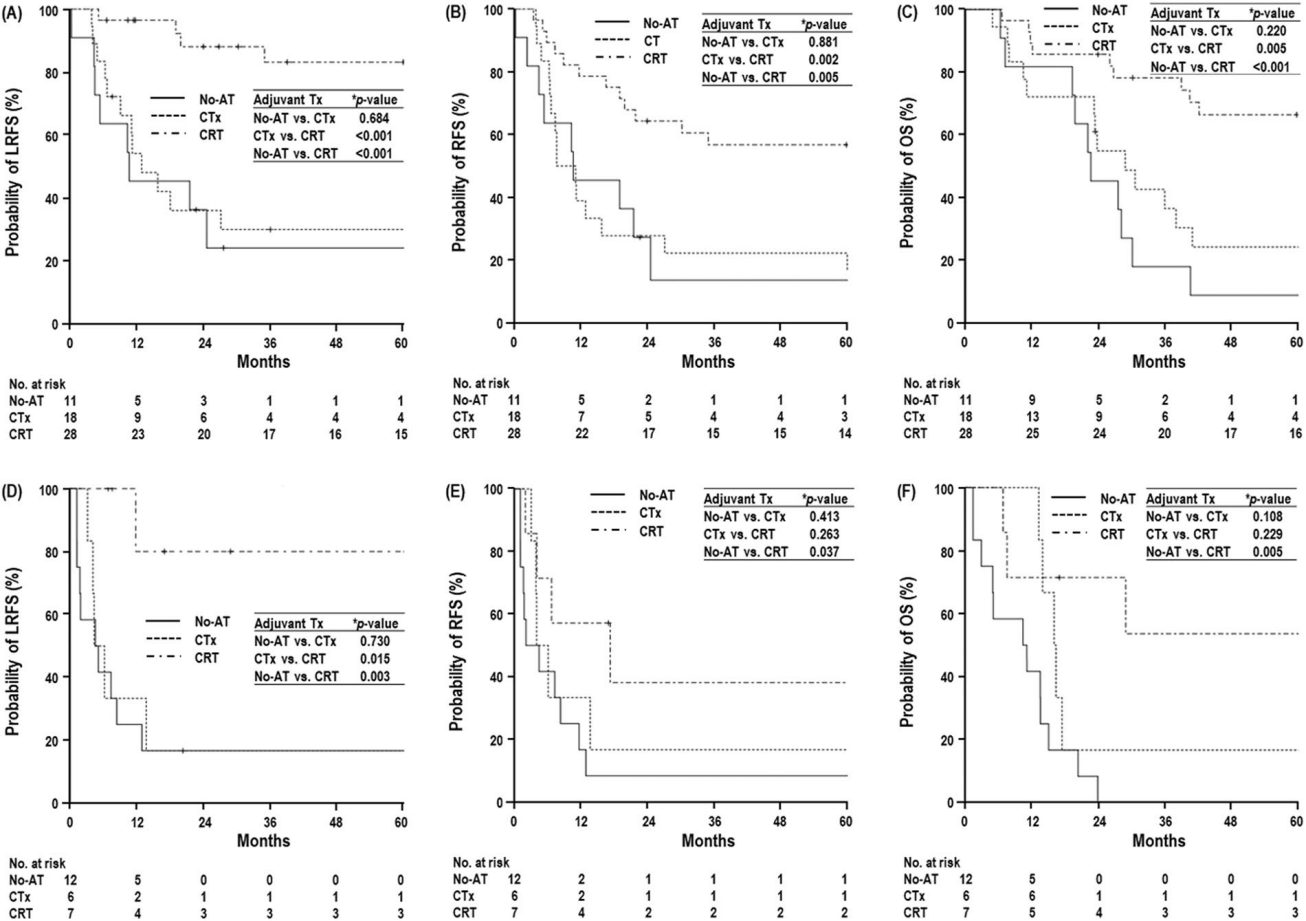
Locoregional recurrence-free survival (LRFS), relapse-free survival (RFS), and overall survival (OS) curves according to adjuvant treatments in patients at the T2-3N1M0 stage (**A**–**C**, respectively) and T2-3N2M0 stage (**D–F**, respectively). Abbreviations: same as in Fig. 1. ^*^Log-rank test.

Univariate analyses for LRFS, RFS and OS are summarized in Table 2 and Fig. 2. In patients at the T2-3N0M0 stage, the CRT group exhibited higher trends in the 5-year LRFS, RFS and OS rates than the CTx and No-AT groups, but these differences were not significant (*p* > 0.05 each) (Table 2) (Fig. 2A–C). In patients at the T2-3N1-2M0 stage, the CRT group had significantly higher 5-year LRFS, RFS and OS rates than the CTx and No-AT groups (*p* > 0.05 each) (Table 2) (Fig. 2D–F), and the CTx group exhibited higher trends in 5-year LRFS, RFS and OS rates than the No-AT group (Table 2) (Fig. 2D–F). In the multivariate analysis, the use of adjuvant therapy was not a significant factor in LRFS, RFS and OS in patients at the T2-3N0M0 stage, but it was a significant factor in LRFS, RFS and OS in patients at the T2-3N1-2M0 stage. N classification and postoperative CA 19-9 level, together with the use of adjuvant therapy, were also significant factors in all LRFS, RFS and OS in the multivariate analysis (*p* < 0.05 each) (Table 3). In patients at the T2-3N1-2M0 stage, the postoperative CA 19-9 level was not significantly different among the No-AT, CTx, and CRT groups, but the distribution of the N classification was significantly different among the No-AT, CTx, and CRT groups (Table 1). Thus, to avoid the effect of N classification, LRFS, RFS and OS according to the use of adjuvant therapy (No-AT, CTx and CRT) were compared in the subgroups with T2-3N1M0 and T2-3N2M0 stage disease. In the subgroup with T2-3N1M0, the CRT group had significantly higher LRFS, RFS and OS than the CTx and No-AT groups (*p* < 0.05 each) (Fig. 3A–C) (Supplementary Table 1). In the subgroup with T2-3N2M0, LRFS and RFS were significantly higher in the CRT group than in the CTx and No-AT groups (*p* < 0.05 each) (Fig. 3D,E), and the CRT group had a higher OS than the CTx and No-AT groups, respectively. The difference between the CRT and No-AT groups was significant (*p* < 0.05), but the difference between the CRT and CTx groups was not significant (*p* > 0.05) (Fig. 3F) (Supplementary Table 1).

**Table 2:** Univariate analysis of locoregional recurrence-free survival (LRFS), relapse-free survival (RFS), and overall survival (OS) in patients with (i) T2-3N0M0 and (ii) T2-3N1-2M0 stage disease.

Characteristic		(i) T2-3N0M0						(ii) T2-3N1-2M0﻿					
		LRFS		RFS		OS		LRFS﻿		RFS﻿		OS﻿	
		5 yr, % (95% CI)	^*^ *p*	5 yr, % (95% CI)	^*^ *p*	5 yr, % (95% CI)	^*^ *p*	5 yr, % (95% CI)	^*^ *p*	5 yr, % (95% CI)	^*^ *p*	5 yr, % (95% CI)	^*^ *p*
Gender	Male	77.3 (62.2–92.4)	0.970	73.4 (58.3–88.5)	0.943	72.1 (56.4–87.8)	0.382	49.3 (30.5–68.1)	0.793	37.4 (20.9–53.9)	0.297	42.3 (25.2–59.4)	0.340
	Female	78.7 (64.6–92.8)		72.1 (56.4–87.8)		81.2 (67.5–94.9)		46.6 (31.5–61.7)		26.4 (13.7–39.1)		29.7 (16.4–43.0)	
Age (years)	≤60	78.2 (59.2–97.2)	0.810	72.9 (52.5–93.2)	0.757	89.5 (75.8–103.2)	0.113	52.5 (33.7–71.3)	0.456	39.3 (21.3–57.3)	0.184	42.1 (23.5–60.7)	0.056
	>60	78.1 (65.9–90.3)		72.8 (60.1–85.5)		71.4 (58.1–84.7)		44.8 (30.3–59.3)		26.1 (13.9–38.3)		31.1 (18.4–43.8)	
Tumor size^*^ (cm)	≤3	91.2 (81.8–100.6)	0.018	85.2 (73.2–97.2)	0.021	88.3 (77.5–99.1)	0.060	55.6 (38.4–72.8)	0.083	34.5 (19.2–49.8)	0.425	41.7 (25.8–57.6)	0.375
	>3	62.9 (45.1–80.7)		58.8 (41.4–76.2)		64.3 (47.1–81.5)		40.8 (25.7–55.9)		27.9 (14.5–41.2))		28.4 (14.5–42.3)	
T classification	T2	93.3 (86.0–100.6)	<0.001	87.1 (77.5–96.7)	<0.001	86.8 (76.8–96.8)	<0.001	56.3 (40.4–72.2)	0.099	39.6 (24.5–54.7)	0.074	43.5 (28.2–58.7)	0.086
	T3	34.6 (10.5–58.7)		33.3 (11.5–55.1)		48.6 (24.9–72.3)		38.9 (22.8–55.0)		21.8 (8.9–34.7)		25.4 (11.5–39.3)	
N classification	N1	—	—	—	—	—	—	54.6 (40.9–68.3)	0.002	36.0 (23.3–48.7)	0.002	42.0 (28.9–55.1)	<0.001
	N2	—		—		—		32.9 (13.5–52.3)		19.2 (3.3–35.1)		18.0 (2.3–33.7)	
Preop CA 19-9 level	≤37	86.7 (77.5–95.9)	<0.001	81.4 (70.8–92.0)	<0.001	80.7 (69.7–91.7)	0.049	55.2 (40.9–69.5)	0.003	35.2 (22.3–48.1)	0.020	41.4 (27.9–54.9)	0.104
(U/mL)	>37	35. 0 (4.8–65.2)		30.0 (2.6–57.4)		56.2 (27.2–85.2)		32.1 (14.1–50.1)		22.2 (6.5–37.9)		22.2 (6.5–37.9)	
Postop CA 19-9 level	≤37	82.4 (72.4–92.3)	0.001	78 (67.4–88.6)	0.001	80.8 (70.4–91.2)	0.009	54.9 (41.8–68.0)	<0.001	38.5 (26.0–51.0)	<0.001	42.4 (29.9–54.9)	0.001
(U/mL)	>37	^†^28.6 (−15.1–72.3)		^†^19.0 (−13.9–51.9)		35.7 (−3.1–74.5)		23.7 (3.1–44.3)		5.3 (−4.7–15.3)		10.5 (−3.2–24.2)	
Histologic	WD/MD	81.8 (70.2–93.4)	0.222	76.2 (60.7–91.7)	0.272	80.1 (68.3–91.9)	0.410	53.0 (38.9–67.1)	0.181	36.3 (22.8–49.8)	0.119	40.3 (26.4–54.2)	0.159
differentiation	PD	67.5 (45.9–89.1)		63.2 (41.4–85.0)		67.7 (46.3–89.1)		38.6 (19.6–57.6)		22.1 (7.2–37.0)		25.8 (10.3–41.2)	
Resection margin	Negative	78.9 (68.7–89.1)	0.522	73.2 (62.2–84.2)	0.579	77.7 (67.3–88.1)	0.467	48.0 (35.7–60.3)	0.848	31.6 (21.2–42.0)	0.243	34.9 (24.3–45.4)	0.837
	Close	^‡^50.0 (−19.4–119.4)		^‡^66.7 (13.3–120)		0 (-)		^†^50.0 (−19.4–119.4)		^†^0 (−)		^†^50 (−19.4–119.4)	
Vascular invasion	No	83.5 (73.5–93.5)	<0.001	78.7 (67.9–89.5)	<0.001	83.8 (74.0–93.6)	<0.001	64.7 (49.2–80.2)	0.002	51.4 (35.1–67.7)	<0.001	58.8 (42.7–74.9)	<0.001
	Yes	^†^37.0 (0.3–73.4)		^†^33.3 (2.5–64.1)		^†^26.7 (−4.5–57.9)		30.2 (14.5–45.9)		13.0 (2.8–23.2)		12 (2.2–21.8)	
Lymphatic invasion	No	87.3 (76.9–97.7)	0.002	82.9 (71.4–94.5)	0.001	89.8 (80.2–99.4)	<0.001	82.2 (64.0–100.4)	0.004	60.2 (37.3–83.1)	0.005	59.3 (36.0–82.6)	0.042
	Yes	62.0 (42.2–81.8)		54.7 (34.9–74.5)		53.3 (32.5–74.1)		37.5 (24.8–50.2)		22.4 (12.0–32.8)		27.9 (16.5–39.3)	
Perineural invasion	No	90.6 (81.8–99.4)	0.001	85.7 (75.1–96.3)	0.001	90.5 (81.7–99.3)	0.001	65.2 (47.6–82.8)	0.026	45.7 (27.7–63.7)	0.017	51.6 (33.4–69.8)	0.023
	Yes	53.1 (31.1–75.1)		48.2 (27.4–69.0)		51.2 (29.6–72.8)		36.2 (21.9–50.5)		22.4 (10.8–34.0)		25.1 (12.9–37.3)	
Adjuvant Therapy	No-AT	79.0 (64.1–93.9)	0.354	73.2 (57.3–89.1)	0.670	74.8 (58.3–91.3)	0.849	19.0 (1.8–36.2)	<0.001	11.6 (−2.3–25.5)	<0.001	4.3 (−4.1–12.7)	<0.001
	CTx	65.0 (40.1–89.9)		65.5 (40.8–90.2)		73.3 (51.0–95.6)		26.8 (8.6–45.0)		16.7 (1.8–31.6)		22.7 (5.4–39.9)	
	CRT	86.2 (71.7–100.7)		75.0 (55.0–95.0)		81.1 (63.9–98.3)		82.1 (67.8–96.4)		53.3 (36.4–70.2)		64 (47.5–80.5)	

Abbreviations: yr, year; CI, confidence interval; others are the same as in Table 1.

^*^log-rank test.

^†^1-year survival rate.

**Table 3:** Multivariate analysis of locoregional recurrence-free survival (LRFS), relapse-free survival (RFS), and overall survival (OS) in patients with (i) T2-3N0M0 and (ii) T2-3N1-2M0 stage disease.

Factor		LRFS		RFS		OS	
		HR (95% CI)	^*^ *p*	HR (95% CI)	^*^ *p*	HR (95% CI)	^*^ *p*
**(i) T2-3N0M0**							
T classification	T2	1.000		1.000		—	
	T3	13.341 (4.098–43.433)	0.001	8.351 (3.204–21.763)	<0.001	—	—
Vascular invasion	No	—		—		1.000	
	Yes	—	—	—	—	5.703 (1.964–16.561)	0.001
Perineural invasion	No	—		—		1.000	
	Yes	—	—	—	—	3.398 (1.308–8.825)	0.012
**(ii) T2-3N1-2M0**							
N classification	N1	1.000		1.000		1.000	
	N2	3.232 (1.591–6.566)	0.001	2.621 (1.388–4.948)	0.003	2.232 (1.218–4.091)	0.009
Postop CA 19-9 level	≤37	1.000		1.000		1.000	
(U/mL)	>37	4.167 (1.929–9.001)	0.001	4.776 (2.366–9.641)	<0.001	2.248 (1.215–4.160)	0.016
Vascular invasion	No	—		1.000		1.000	
	Yes	—	—	3.258 (1.384–7.668)	0.007	2.953 (1.620–5.383)	<0.001
Lymphatic invasion	No	1.000		—		—	
	Yes	5.819 (1.801–18.798)	0.003	—	—	—	—
Adjuvant Therapy	No-AT	1.000		1.000		1.000	
	CTx	0.360 (0.170–0.760)	0.007	0.409 (0.203–0.826)	0.013	0.328 (0.165–0.651)	0.001
	CRT	0.081 (0.029–0.228)	<0.001	0.247 (0.123–0.497)	<0.001	0.130 (0.060–0.282)	<0.001

Abbreviations: HR, hazard ratio; CI, confidence interval; others are the same as in Table 1.

^*^Multivariate analysis using the Cox proportional hazards model.

## Discussion

As in other malignancies, disease recurrence after radical resection for GB cancer inevitably leads to death. However, little information is available regarding the patterns of disease recurrence in resected GB cancer patients. Several previous studies have reported a 31.9–66.3% disease recurrence rate: 33.9–79.2%, 52.6–79.8% and 18.9–50% disease recurrence occurred at locoregional sites, distant sites, and both, respectively^1–6^, and the most common sites of disease recurrence were regional lymph nodes (27.8–47.3%), the liver (22.2–36.8%), and the local area (20.8–28.3%)^1,2,5^. These data suggested that locoregional and distant recurrences in patients with resected GB cancer were common and that adjuvant treatment should be considered in resected GB cancer patients to reduce both locoregional and distant recurrence. In the present study, disease recurrence rates in resected GB cancer patients at the T2-3N0M0 and T2-3N1-2M0 stages were still relatively high (27.5% and 68.3%, respectively). Although the incidences of locoregional recurrence and distant recurrence in resected GB cancer patients with T2-3N0M0 stage disease were not significantly different among the No-AT, CTx, and CRT groups (*p* > 0.05), the incidences of locoregional recurrence and distant recurrence were significantly different among the No-AT, CTx, and CRT groups in patients at the T2-3N1-2M0 stage (*p* < 0.05) (Fig. 1). These findings suggest that CRT reduces both locoregional and distant recurrence and subsequently improves survival. However, the incidence of distant recurrence was still high (42.8%) in resected GB cancer even after CRT, and the addition of CTx to CRT might be considered to further reduce distant recurrence.

Through randomized phase III trials evaluating the adjuvant CTx using 5-FU and mitomycin C for pancreatobiliary cancer, which included patients with stage IV and/or received surgical resection with non-curative intent, Takada *et al*.^16^, showed that the 5-year OS rate in the subgroup with GB cancer (n = 112) was significantly superior in patients who received adjuvant CTx than those who did not (26% vs. 14.4%, *p* < 0.05). A recent phase III trial^24^ for resected biliary cancer that included 79 GB cancer cases showed that adjuvant capecitabine significantly improved the median survival time (53 months vs. 36 months, *p* = 0.028) compared with that in the per-protocol population, but it did not significantly improve survival in the intention-to-treat population. However, these studies did not compare CTx with CRT in patients with resected GB cancer. Another recent multi-institutional study^11^ of GB cancer patients (n = 291) undergoing surgical resection with curative intent showed that adjuvant treatments, such as CTx and CRT, improved the disease-free survival and OS, and its benefit was pronounced in patients with adverse prognostic features, such as advanced tumor stage, lymph node metastasis and a microscopic positive surgical margin. In addition, in an analysis of resected GB cancer from the Surveillance, Epidemiology, End Results (SEER) database, Wang *et al*.^15^ showed that adjuvant therapy yielded a survival benefit in ≥T2 and lymph node-positive patients and that CRT provided a greater advantage than CTx alone. Similar to the above studies, the present study showed that compared with CTx and No-AT, CRT did not significantly improve the LRFS, RFS and OS in resected GB cancer patients with lymph node-negative T2-3 disease, but did significantly improve the LRFS, RFS and OS in resected GB cancer patients with lymph node-positive disease (*p* < 0.05 each) (Table 2) (Fig. 2D–F).

This study had inherent limitations stemming from single institutional retrospective data with heterogeneity of treatments, such as heterogeneous chemotherapeutic regimens, completeness of surgery, etc., and thus possible selection bias was not thoroughly assessed. Nevertheless, to date, the present investigation was a relatively large study (n = 151) in GB cancer patients who received curative-intent surgical resection for investigating the benefit of CRT compared with CTx alone and observation. We compared the clinicopathological factors, patterns of failure, LRFS, RFS, and OS among three groups (CRT, CTx, No-AT) according to tumor stage, i.e., T2-3N0M0 (n = 69) and T2-3N1-2M0 (n = 82), and showed that compared with CTx and No-AT, CRT yielded significant benefits in LRFS, RFS and OS in resected GB patients with T2-3N1-2M0 stage disease. Although, in resected GB cancer patients at the T2-3N0M0 stage, adjuvant treatments, such as CRT and CTx, did not show a significant improvement in LRFS, RFS and OS compared with that yielded by No-AT, further investigation to develop effective adjuvant treatment strategies should be conducted due to the relatively high rate (25–33.5%) of disease recurrence.

The present study showed that the disease recurrence in resected GB cancer at the T2-3N0M0 and T2-3N0M0 stage was as high as 26.7% and 91.3%, respectively, in patients who did not undergo adjuvant treatment, suggesting that effective adjuvant treatment might be needed for these patients. In addition, our data showed that adjuvant CRT could significantly improve the LRFS, RFS and OS in resected GB cancer patients with T2-3N1-2M0 stage disease and suggest that further large-scale studies for investigating the optimal adjuvant treatment strategies, such as CRT, CTx using new chemotherapeutic regimens, and combination of CRT and CTx, are needed.

## Methods

### Patients

Between October 2001 and October 2017, patients who received surgical resection for primary GB carcinoma were registered, and the database was reviewed. Medical records, including surgeon’s notes, radiologic imaging, surgical and pathological reports, and discharge summaries, of each patient were evaluated, and clinicopathological data, such as age, gender, histology, grade, carbohydrate 19-9 (CA 19-9) antigen, pathologic stage, surgical method, and adjuvant therapy were obtained. Pathologic stage was determined by the American Joint Committee on Cancer (AJCC) staging system (8th edition). The inclusion criteria for the present study were as follows: (i) histologically confirmed adenocarcinoma of the gallbladder; (ii) surgical resection without positive resection margins or gross residual disease; (iii) T2 or higher disease; (iv) no distant metastasis; and (v) no other previous or current malignancy. This study was approved by the Institutional Review Board (IRB) of the National Cancer Center (NCC) in Korea (NCC20170148), and all methods were performed in accordance with the relevant guidelines and regulations. The IRB of NCC waived the need for informed consent because of the retrospective nature of this research.

### Evaluation and statistical considerations

Recurrence was proven by pathologic and/or radiologic findings showing an increase in size over time. Locoregional and distant recurrence was defined as a tumor recurrence within resection margins and regional lymph nodes (porta hepatis, peripancreatic, celiac, origin of the superior mesenteric artery and para-aortic nodes) and the development of new tumors in discontinuous liver, peritoneum, lung, or distant lymph nodes, respectively. Overall survival (OS), recurrence-free survival (RFS) and locoregional recurrence-free survival (LRFS) were estimated from the date of surgical resection to the date of death or last follow-up, any recurrence and locoregional recurrence, respectively. The distributions of clinicopathologic characteristics among subgroups according to the use of adjuvant therapy (No-AT, CTx and CRT) were compared using Fisher’s exact test or one-way analysis of variance. The probability of survival was calculated using the Kaplan-Meier method, and in the univariate analysis, the log-rank test was used to evaluate the effects of the factors on survival. Multivariate analysis was performed using Cox’s proportional hazard model with a stepwise forward selection procedure. Differences in the *p* values of <0.05 were considered statistically significant, and variables with *p* < 0.1 in the univariate analysis were entered into the multivariate analysis. All statistical analyses were performed using STATA software (version 14.0; StataCorp., College Station, TX, USA).

## Supplementary information

Table 1-SI
