# Supplementary material for: Benefit of Adjuvant Chemoradiotherapy in Resected Gallbladder Carcinoma
Source: Sci Rep. 2019 Aug 13;9:11770. doi: 10.1038/s41598-019-48099-z (PMC6692378; doi:10.1038/s41598-019-48099-z)
Supplement: Supplementary file 1 — Table 1-SI [file 41598_2019_48099_MOESM1_ESM.doc]

**Supplementary Information**

**Benefit of Adjuvant Chemoradiotherapy in Resected Gallbladder Carcinoma**

Tae Hyun Kim^1,2^, Sang Myung Woo^1^, Woo Jin Lee^1^, Eun Sang Oh^2^, Sang Hee Youn^2^, Sung Ho Moon^2^, Sang Soo Kim^2^, Sung Sik Han^1^, Sang-Jae Park^1^, Dae Yong Kim^2^

^1^Center for Liver and Pancreatobiliary Cancer, Research Institute and Hospital, National Cancer Center, Goyang, 10408, Korea

^2^Center for Proton Therapy, Research Institute and Hospital, National Cancer Center, Goyang, 10408, Korea

Table 1-SI. locoregional recurrence-free survival (LRFS), relapse-free survival (RFS), and overall survival (OS) according to the use of adjuvant therapy in patients with (i) T2-3N1M0 and (ii) T2-3N2M0 Stage

| (i) T2-3N1M0 |  |  |  |
| --- | --- | --- | --- |
|  | LRFS | RFS | OS |
| Adjuvant therapy | 5 yr, % (95% CI) | 5 yr, % (95% CI) | 5 yr, % (95% CI) |
| No-AT | 24.2 ( 8.1 – 51.2) | 13.6 (-9.3 – 36.5) | 9.1 (-8.0 – 26.2) |
| CTx | 30.1 (8.1 – 52.1) | 16.7 (-0.5 – 33.9) | 22.4 (1.8 – 43) |
| CRT | 83.2 (67.9 – 94.5) | 56.7 (38.3 – 75.1) | 66.5 (48.7 – 84.3) |
|  |  |  |  |
| (ii) T2-3N2M0 |  |  |  |
|  | LRFS | RFS | OS |
| Adjuvant therapy | 5 yr, % (95% CI) | 5 yr, % (95% CI) | 5 yr, % (95% CI) |
| No-AT | 16.7 (-4.5 – 37.9) | 13.6 (-9.3 – 36.5) | 0 ( - ) |
| CTx | 13.8 (-16.0 – 43.6) | 16.7 (-0.5- 33.9) | 16.7 (-13.1 – 46.5) |
| CRT | 80.0 (44.9 – 115.1) | 38.1 (-0.9 – 77.1) | 35.7 (-3.1 – 74.5) |

Abbreviations: No-AT, no adjuvant therapy; CTx, chemotherapy; CRT, chemoradiotherapy; yr, year; and CI, confidence interval.
